# Indirect Rotavirus Vaccine Effectiveness for the Prevention of Rotavirus Hospitalization: A Systematic Review and Meta-Analysis

**DOI:** 10.4269/ajtmh.17-0705

**Published:** 2018-02-12

**Authors:** Katherine L. Rosettie, Theo Vos, Ali H. Mokdad, Abraham D. Flaxman, Ibrahim Khalil, Christopher Troeger, Marcia R. Weaver

**Affiliations:** Institute for Health Metrics and Evaluation, University of Washington, Seattle, Washington

## Abstract

Two rotavirus vaccines, RotaTeq and Rotarix, are licensed for global use; however, the protection they confer to unvaccinated individuals through indirect effects remains unknown. We systematically reviewed the literature and quantified indirect rotavirus vaccine effectiveness (VE) for preventing rotavirus hospitalization in children aged less than 5 years. From 148 identified abstracts, 14 studies met our eligibility criteria. In our main analysis using a random-effects model, indirect rotavirus VE was 48% (95% confidence interval [CI]: 39–55%). In a subgroup analysis by country income level, indirect VE was greater in high-income countries (52%; 95% CI: 43–60%) than in low- and middle-income countries (LMICs) (25%; 95% CI: 5–41%). In a sensitivity analysis using a quality-effects model, the indirect VE in LMICs was not statistically significant (25%; 95% CI: 0–44%). Our findings highlight the importance of increasing rotavirus vaccine coverage, particularly in LMICs where evidence for indirect VE is limited and rotavirus burden is high.

Diarrheal diseases are among the top five leading causes of child mortality globally.^1^ In particular, rotavirus is the most common cause of child mortality because of diarrhea, leading to an estimated 146,000 deaths in children aged less than 5 years in 2015.^2^ To address this burden, the World Health Organization recommends the inclusion of rotavirus vaccines in all national immunization programs.^3^

Two live-attenuated oral rotavirus vaccines are licensed for global use and have been introduced in over 100 countries since 2006,^4^ including a pentavalent vaccine (RotaTeq [RV5]) administered at 2, 4, and 6 months of age and monovalent vaccine (Rotarix [RV1]) administered at 2 and 4 months of age.

Rotavirus vaccine effectiveness (VE) can be divided into two major components: direct VE and indirect VE. Direct rotavirus VE has been quantified in previous meta-analyses^5–7^ and is measured by comparing the risk of infection in vaccinated versus unvaccinated individuals. Less is known about indirect rotavirus VE, which is defined as the population-level effect of widespread vaccination in unvaccinated individuals (sometimes called the “herd effect”).^8^ Indirect rotavirus VE is measured by comparing the risk of rotavirus infection in unvaccinated individuals living in populations with and without rotavirus vaccine coverage.

Although evidence for indirect rotavirus VE has been summarized in previous reviews,^9,10^ it has not been formally quantified in children aged less than 5 years. We systematically reviewed and quantified indirect rotavirus VE for preventing rotavirus hospitalization in this age group, incorporating newly available data that were not captured in previous reviews.

We searched multiple online databases in June 2017, including PubMed, EMBASE, and Web of Science using combinations of the following search terms: herd immunity, adaptive immunity, herd effect, herd protection, indirect effect, indirect effectiveness, indirect immunity, rotavirus, rotavirus vaccine, immunization, RV5, and RV1. We also hand-searched the reference lists of identified articles for additional relevant studies.

We included all experimental and quasi-experimental studies in any setting that reported the incidence of rotavirus hospitalization in unvaccinated children aged less than 5 years in populations with and without rotavirus vaccine coverage. We excluded commentaries, reviews, modeling studies, and phase I and phase II trials. Studies were also excluded if they did not report relative risks (RRs) of rotavirus hospitalization and their uncertainty (e.g., 95% confidence intervals [CIs]) or did not report enough raw data to calculate both.

For each study, we extracted the following data: author names, publication date, country, study design, vaccine coverage, study period, age range of subjects, year of vaccine introduction, vaccine type (RV1 and RV5), and indirect VE estimates and their uncertainty for all reported outcomes in unvaccinated populations.

For studies that reported outcomes in multiple age groups measured at the same time point, we treated each outcome as a separate effect estimate in our meta-analysis.^11^ For example, effect estimates for infants younger than 12 months and 12–24 months old in 2007 from the same study would be included separately in our meta-analysis. For studies reporting outcomes from subgroups that were followed over time, we calculated the mean effect size and its variance to include as a single estimate in our meta-analysis.^11^ For example, effect estimates in 3-year-olds in 2007, 4-year-olds in 2008, and 5-year-olds in 2009 from the same study would be averaged to produce a single pooled estimate.

The primary outcome was the RR of rotavirus hospitalization in unvaccinated children in populations with and without rotavirus vaccination. These populations were either separated geographically (i.e., different communities) or temporally (i.e., prevaccine and postvaccine introduction).

For our main analysis, we used an inverse-variance–weighted random-effects model. We assessed heterogeneity using Cochran’s Q test and *I*^2^, and we explored potential sources of heterogeneity through subgroup analysis and meta-regression. All random-effects analyses were conducted using Stata 13.0 (Stata Corp., College Station, TX).^12^

In sensitivity analyses, we used quality-effects models to assess the effects of study quality on our pooled estimates.^13^ We developed a five-point quality scoring scheme, with points awarded based on study design, assessment of vaccine coverage, control for confounders, and evidence of selection bias (Supplemental Table 1). All quality-effects analyses were conducted using MetaXL version 5.3 in Microsoft Excel (Microsoft Corp., Redmond, WA).^14^

We screened 148 unique titles and abstracts identified through our systematic literature search (Figure 1). A total of 58 full-text articles were reviewed, and 14 articles met our prespecified eligibility criteria. These included 13 pre-post quasiexperimental studies^8,15–26^ and one cluster-randomized trial.^27^ Ten studies were conducted in high-income countries,^8,15,17–19,21–25^ and four were conducted in low- and middle-income countries (LMICs).^16,20,26,27^ Most studies measured outcomes only in age-ineligible unvaccinated children (*N* = 9),^15–22,26^ two studies measured outcomes in both age-eligible and age-ineligible unvaccinated children,^24,27^ and three studies measured outcomes only in age-eligible unvaccinated children.^8,23,25^ Partial rotavirus vaccine coverage (at least one dose) was reported in 13 studies and ranged from 35% to 89.6%,^8,15–21,23–27^ with most studies reporting partial coverage above 70% (*N* = 9) (Figure 2).^15–20,23,24,27^ Complete dose coverage was reported by three studies and ranged from 50% to 93%.^16,17,22^

**Figure 1.**
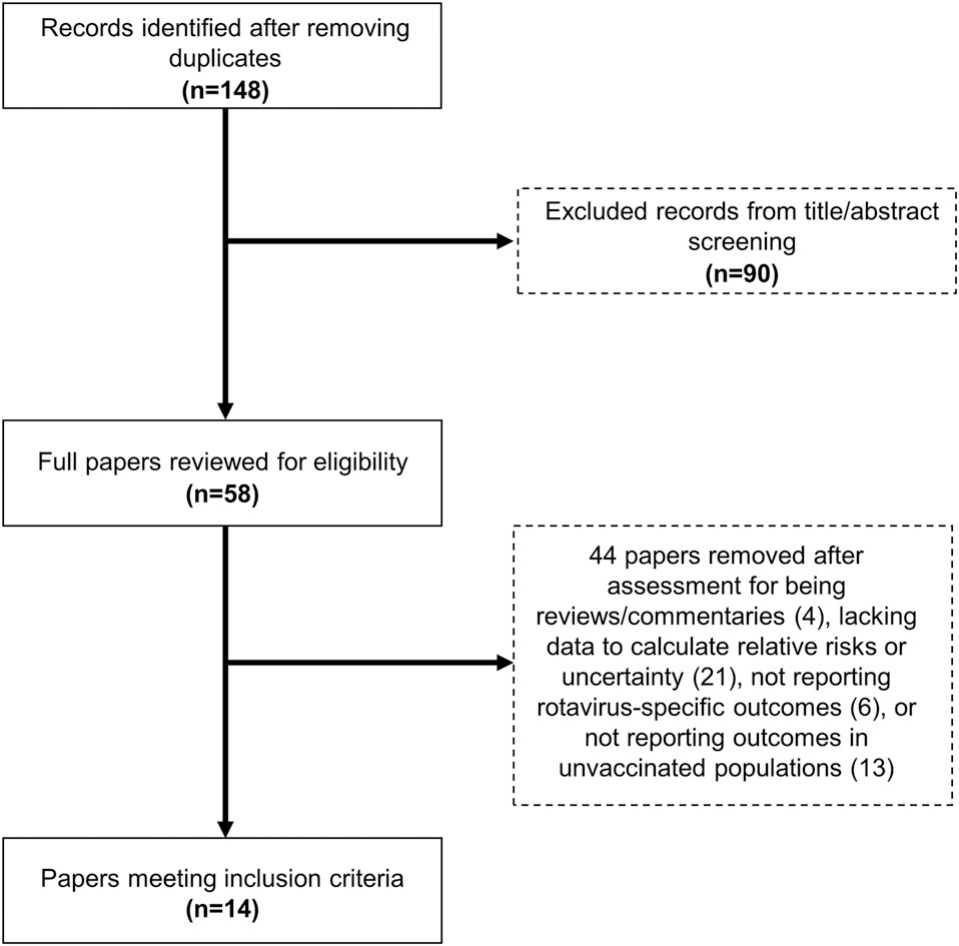
Screening and selection process of interventional and observational studies evaluating the indirect rotavirus vaccine effectiveness for preventing rotavirus hospitalizations in children aged less than 5 years.

**Figure 2.**
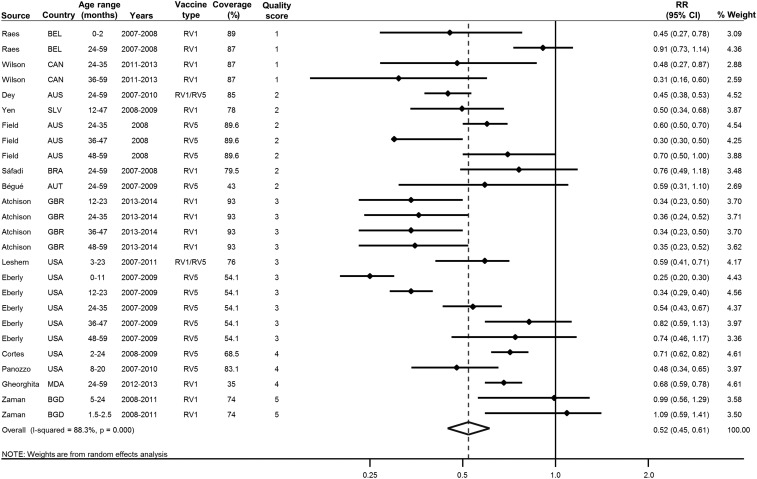
Pooled relative risk (RR) for rotavirus hospitalization comparing unvaccinated children in populations with and without rotavirus vaccination. Effect sizes were pooled using inverse-variance–weighted random-effects model. Separate estimates from individual studies were included if they were derived from independent subgroups. If multiple estimates reported from the same study were not independent, the mean estimates were used in our pooled meta-analysis. Coverage is defined as the percentage of the population that received at least one dose of rotavirus vaccine. Quality scores were calculated on a five-point continuum based on study design, measurement of vaccine coverage, controlling for confounders, and evidence for selection bias. AUS = Australia; AUT = Austria; BEL = Belgium; BGD = Bangladesh; BRA = Brazil; CAN = Canada; CI = confidence interval; GBR = United Kingdom; MDA = Moldova; NS = not specified; RV1 = Rotarix; RV5 = RotaTeq; SLV = El Salvador; USA = United States of America.

For all analyses, we converted the pooled RR to indirect VE by multiplying 1-RR by 100%. Pooling all studies using an inverse-variance–weighted random-effects model, the indirect rotavirus VE was 48% (95% CI: 39–55%) (Figure 2). In a subgroup analysis comparing the indirect rotavirus VE in high-income countries with LMICs, the pooled estimates were 52% (95% CI: 43–60%) and 25% (95% CI: 5–41%), respectively (Figure 3).

**Figure 3.**
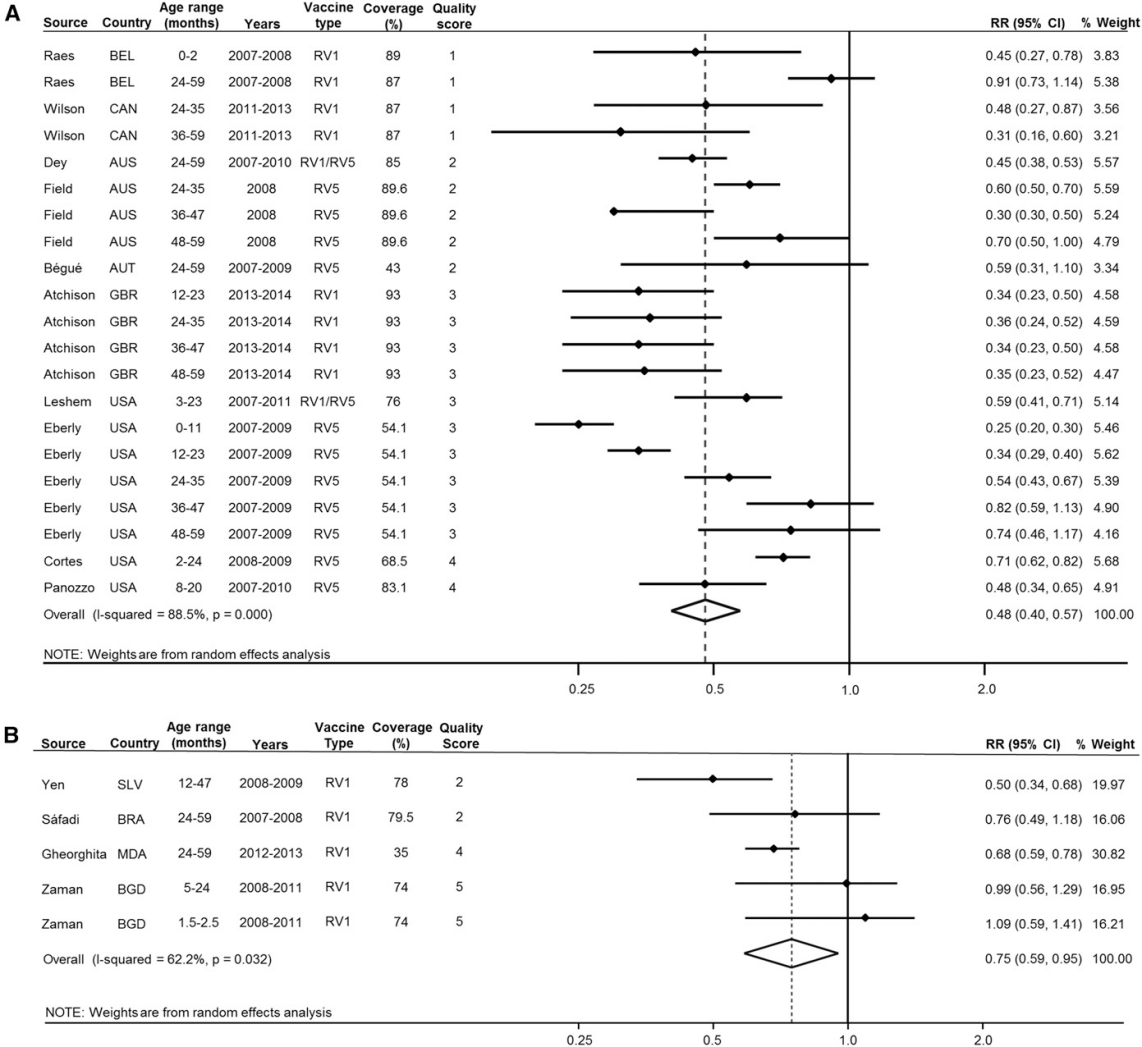
Pooled relative risks (RRs) for rotavirus hospitalization comparing unvaccinated children in populations with and without rotavirus vaccination in high-income countries (**A**) and low- and middle-income countries (**B**). Effect sizes were pooled using inverse-variance–weighted random-effects model. Separate estimates from individual studies were included if they were derived from independent subgroups. If multiple estimates reported from the same study were not independent, the mean estimates were used in our pooled meta-analysis. AUS = Australia; AUT = Austria; BEL = Belgium; BGD = Bangladesh; BRA = Brazil; CAN = Canada; CI = confidence interval; GBR = United Kingdom; MDA = Moldova; RV1 = Rotarix; RV5 = RotaTeq; SLV = El Salvador; USA = United States of America.

For our pooled estimate including all studies, there was considerable heterogeneity (*I*^2^ = 88.3%) (Figure 2). In univariate meta-regression, the country income level was a statistically significant source of heterogeneity (*P* = 0.022; Supplemental Table 2). In additional meta-regressions, findings were not significantly different according to the median age of participants, vaccine coverage, age eligibility, or study quality score (Supplemental Table 2).

In a sensitivity analysis using a quality-effects model, the pooled effect size including all studies was 46% (95% CI: 34–55%) (Supplemental Figure 1). In a subgroup analysis by country income level using a quality-effects model, the pooled effect in LMICs was no longer statistically significant (25%; 95% CI: 0–44%), whereas there was a 51% indirect VE in high-income countries (95% CI: 40–61%) (Supplemental Figures 2 and 3).

Our systematic review of the empirical evidence of indirect rotavirus VE demonstrates a protective benefit of the vaccine against rotavirus hospitalization in unvaccinated populations. Although our pooled estimate shows a statistically significant indirect VE across all studies, we found stronger evidence for indirect VE in high-income countries than in LMICs, especially after accounting for study quality.

Our findings of differential indirect VE estimates by country income level may be partially driven by regional variation in direct rotavirus VE, as evidenced by a previous meta-analysis that found a larger direct rotavirus VE in high-income countries than in LMICs.^5^ These regional differences in direct rotavirus VE may be attributed to a number of biological and environmental factors, such as differences in the gut microbiome and access to safe water and sanitation.^28^ In regions with lower direct VE, higher vaccine coverage may be necessary to reach a given level of indirect VE. This phenomenon was seen with the oral polio vaccine, where regional differences in direct VE necessitated higher coverage in South America to achieve the same level of indirect VE as North America.^29^

A limitation in our analysis is that most studies that measured indirect rotavirus VE used pre-post quasi-experimental designs. Secular trends in diarrhea mortality may confound the outcomes in pre-post study designs if these trends are not adequately controlled for. However, it would be unethical to randomize individuals to rotavirus vaccines, given that RV1 and RV5 are efficacious. For future vaccine trials, it will be important to use interventional designs that allow for measurements of both direct and indirect vaccine effects to capture the full benefits of vaccine introduction and scale-up.

Another limitation is that many studies measured coverage using national databases and national surveillance, which may not adequately predict indirect VE because they fail to capture social network dynamics and nonrandom vaccination patterns that influence rotavirus transmission in study populations.^30^ In addition, most studies only measured partial vaccine coverage, yet full dose coverage is likely a more accurate measure of protection.

We included multiple estimates from single studies in our meta-analyses if independence between the estimates was a reasonable assumption, meaning there was no overlap in participants in either the control (“pre”) or intervention (“post”) groups. Although we were conservative in assuming independence, any unmeasured or unreported correlations between estimates that we treated as independent could lead to unit of analysis errors.

Our results support the rapid introduction of the rotavirus vaccine into national immunization programs worldwide, given their proven benefits for vaccinated children^5^ and their potential added benefits for unvaccinated children through their indirect effects. Given the limited evidence for indirect rotavirus VE in LMICs, however, our findings underline the importance of increased efforts to improve rotavirus immunization in these settings with the highest rotavirus burden. Funders, governments, and nongovernmental organizations should work together to increase rotavirus vaccination coverage to meet the Sustainable Development Goals focusing on ending preventable deaths of children aged younger than 5 years and achieving universal coverage of child vaccines by 2030.

## Supplementary Material

Supplemental Figures and Tables
