# Supplementary material for: Indirect Rotavirus Vaccine Effectiveness for the Prevention of Rotavirus Hospitalization: A Systematic Review and Meta-Analysis
Source: Am J Trop Med Hyg. 2018 Feb 12;98(4):1197–201. doi: 10.4269/ajtmh.17-0705 (PMC5928826; doi:10.4269/ajtmh.17-0705)
Supplement: Supplementary file 1 [file tpmd170705.SD1.pdf]

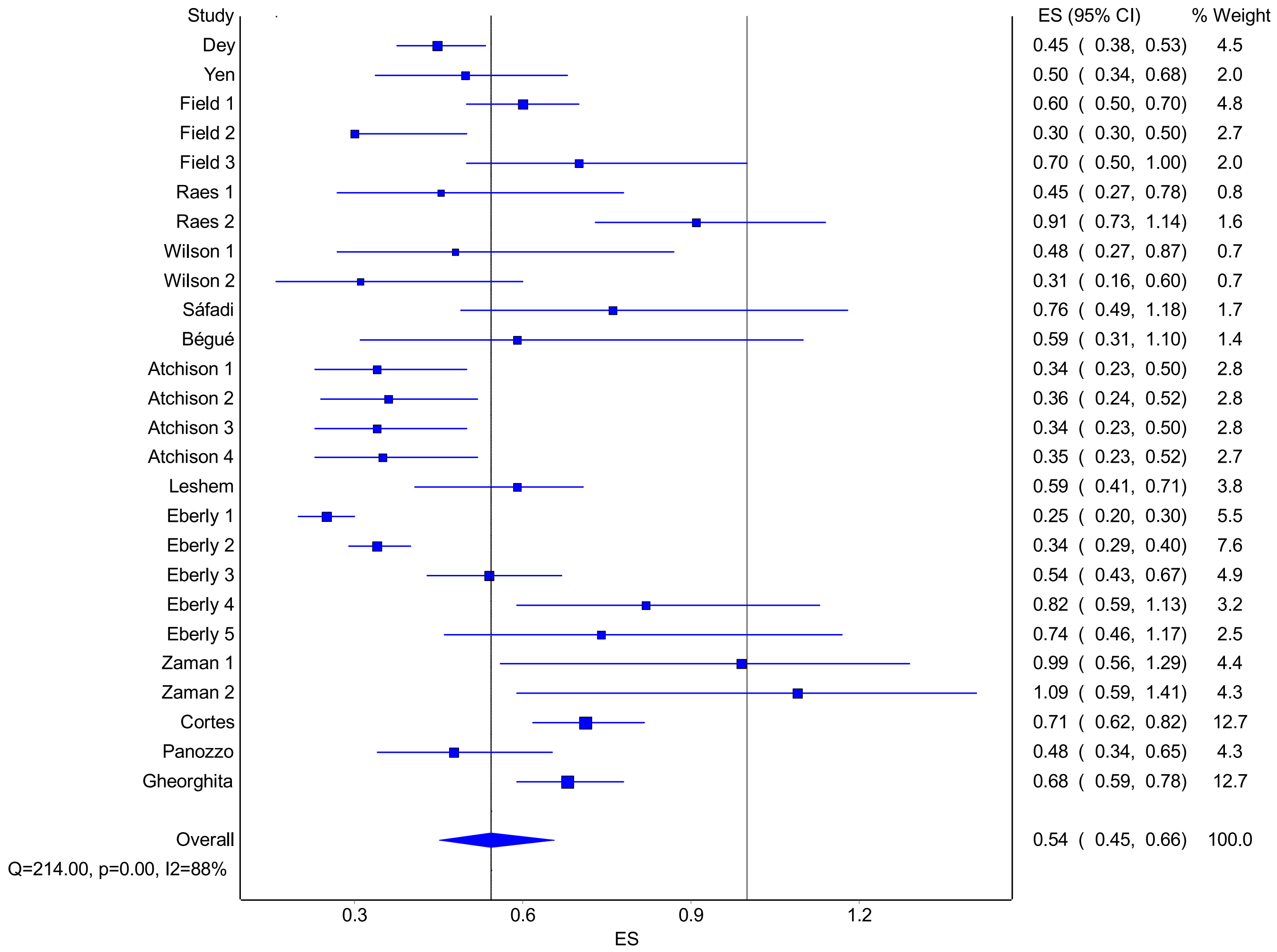

Supplemental Figure 1. Pooled relative risk for rotavirus hospitalization comparing unvaccinated children in populations with and without rotavirus vaccination using a quality-effects model. Separate estimates from individual studies were included if they were derived from independent subgroups. If multiple estimates reported from the same study were not independent, the mean estimates were used in our pooled meta-analysis. Quality scores were calculated on a five-point continuum based on study design, measurement of vaccine coverage, controlling for confounders, and evidence for selection bias. AUS = Australia; AUT = Austria; BEL = Belgium; BGD = Bangladesh; BRA = Brazil; CAN = Canada; CI = confidence interval; GBR = United Kingdom; MDA = Moldova; SLV = El Salvador; USA = United States of America.

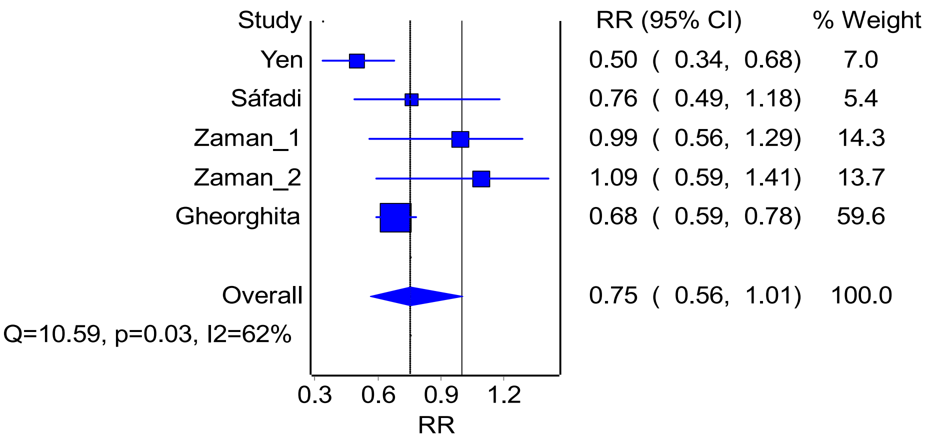

Supplemental Figure 2. Pooled relative risks (RRs) for rotavirus hospitalization comparing unvaccinated children in populations with and without rotavirus vaccination in low- and middle-income countries using a quality-effect model. Separate estimates from individual studies were included if they were derived from independent subgroups. If multiple estimates reported from the same study were not independent, the mean estimates were used in our pooled meta-analysis. Quality scores were calculated on a five-point continuum based on study design, measurement of vaccine coverage, controlling for confounders, and evidence for selection bias. AUS = Australia; AUT = Austria; BEL = Belgium; BGD = Bangladesh; BRA = Brazil; CAN = Canada; CI = confidence interval; GBR = United Kingdom; MDA = Moldova; SLV = El Salvador; USA = United States of America.

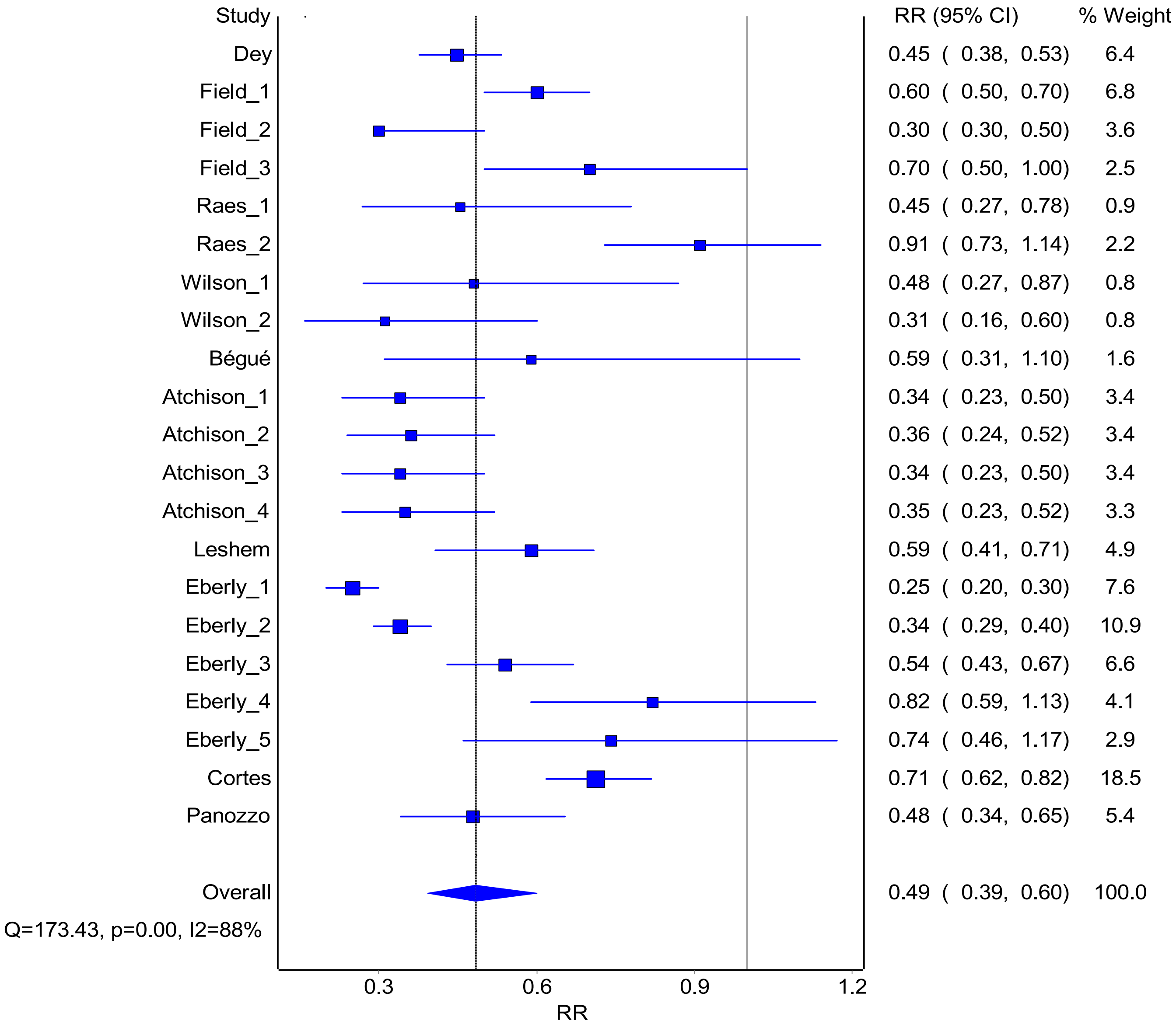

SUPPLEMENTAL FIGURE 3. Pooled relative risks (RRs) for rotavirus hospitalization comparing unvaccinated children in populations with and without rotavirus vaccination in high-income countries using a quality-effects model. Separate estimates from individual studies were included if they were derived from independent subgroups. If multiple estimates reported from the same study were not independent, the mean estimates were used in our pooled meta-analysis. Quality scores were calculated on a five-point continuum based on study design, measurement of vaccine coverage, controlling for confounders, and evidence for selection bias. AUS = Australia; AUT = Austria; BEL = Belgium; BGD = Bangladesh; BRA = Brazil; CAN = Canada; CI = confidence interval; GBR = United Kingdom; MDA = Moldova; SLV = El Salvador; USA = United States of America.

Supplemental Table 1

Quality scoring scheme for individual studies

Criterion	Range		Description
Design	0–1	0	Quasi-experimental
		1	Experimental
Assessment of vaccine coverage	0–2	0	Obtained through the administrative method or vaccine sales data
		1	Obtained from prior studies that measured coverage directly in subpopulations, national immunization databases, or surveillance (not specific to study population)
		2	Obtained from insurance data or medical records of study participants, or other method of active data acquisition
Control for confounding	0–1	0	No control for major confounders, including secular trends
		1	Controlled for secular trends and/or seasonality
Evidence of selection bias	0–1	0	Strong evidence for selection bias
		1	No substantial evidence for selection bias

Supplemental Table 2

Univariate meta-regression models of indirect rotavirus effectiveness by study characteristics

Study characteristic	Number of estimates*	RR (95% CI)	*P* value
Median age of participants	26	1.00 (0.99, 1.01)	0.839
Income level			
High income	21	(Reference)	–
Low- and middle income	5	1.59 (1.08, 2.34)	**0.022**
Age eligibility			
Age ineligible	21	(Reference)	–
Age eligible	5	0.94 (0.64, 1.39)	0.765
Vaccine coverage†	22	1.00 (0.99, 1.01)	0.887
Study quality score‡	26	1.10 (0.94, 1.29)	0.205

CI = confidence interval; RR = relative risks. Bold text indicates statistical significance with a *P* value less than 0.05.

* Estimates were obtained from 14 studies in total. Separate estimates from individual studies were included if they were derived from independent subgroups. If multiple estimates reported from the same study were not independent, the mean estimates were used in our pooled meta-analysis.

† Percent of population that received at least one dose of the rotavirus vaccine.

‡ Study quality score ranges from 0 to 5, with points awarded based on study design, assessment of vaccine coverage, control for confounding, and evidence of selection bias.
